# Effect of 4-Aminopyridine on Action Potential Parameters in Isolated Dog Purkinje Fibers

**DOI:** 10.1111/j.1753-5174.2009.00027.x

**Published:** 2010-03

**Authors:** George Thomas, Brian Klatt, Andrew Blight

**Affiliations:** * Department of Safety Pharmacology, Alcon Research, Ltd.Fort Worth, TX, USA; † Calvert Laboratories, Inc.Olyphant, PA, USA; ‡ Acorda Therapeutics, Inc.Hawthorne, NY, USA

**Keywords:** Fampridine, 4-Aminopyridine, Purkinje Fibers, Action Potential, Cardiac Effects, Torsades de Pointes

## Abstract

**Introduction:**

4-Aminopyridine (fampridine), a potassium channel blocker, has demonstrated efficacy in improving lower extremity strength and walking speed in patients with multiple sclerosis. Since *in vitro* electrophysiologic studies are recommended for evaluating a drug's potential to prolong the QT interval and induce such cardiac arrhythmias as Torsades de Pointes, we examined the electrophysiologic effects of 4-aminopyridine (0.5, 5.0, 50, and 500 µM) on isolated canine Purkinje fibers.

**Methods:**

Microelectrodes monitored the resting membrane potential, overshoot, amplitude of action potential (AP), and maximal rate of depolarization of the AP upstroke in Purkinje fibers stimulated at 0.5 and 1.0 Hz.

**Results:**

None of the above variables were altered in the presence of 4-aminopyridine. The AP duration at 30%, 50%, and 90% repolarization was also monitored, with only the 500-µM concentration at the 1.0-Hz frequency significantly increasing these values with respect to baseline (*P* < 0.05). However, the small sample size (N = 4) was small. The proportional increases, and their 95% confidence intervals, were 90.8% (−36.4%, 218.0%), 25.8% (11.9%, 39.7%), and 22.0% (14.9%, 29.1%) for APD 30%, 50%, and 90% repolarization, respectively. Reverse rate dependence was not observed, suggesting inhibition of ion channels other than those contributing to QT interval prolongation.

## Introduction

A delay in cardiac repolarization induced by non-antiarrhythmic drugs can result in the development of Torsades de Pointes, a potentially fatal cardiac arrhythmia that has led to the withdrawal of several drugs from the market [1,2]. Since prolongation of the cardiac QT interval is a frequent precursor of Torsades de Pointes, QT interval prolongation is recognized as a surrogate marker for a drug's proarrhythmic risk [1,3]. Assessment of the QT interval is recommended by the International Conference on Harmonisation [4] and by the US Food and Drug Administration for new drugs, as well as for approved drugs when new indications, dosages, or routes of administration have been introduced [3]. However, since clinical assessment of QT interval is considered to be a less than optimal method for identifying cardiac risk [1,3], nonclinical methods to assess the human ether-à-go-go related gene (hERG) channel inhibition, prolongation of action potential (AP) and/or QT prolongation in experimental animals are recommended as part of the preclinical evaluation process [5].

Many of the drugs associated with QT prolongation and induction of Torsades de Pointes inhibit hERG [6,7], the gene that encodes the rapid delayed rectifier potassium current (I_Kr_) that is responsible for repolarization of the cardiac myocyte ventricular AP [2,8,9]. However, the electrophysiology of the ventricular AP involves the function of other cardiac ionic currents that could potentially modulate the effects of hERG blockade. Consequently, evaluation of AP parameters complements other techniques for assessing a drug's potential for cardiac toxicity.

Cardiac Purkinje fibers are physiologically appropriate for these types of studies, since these fibers conduct cardiac impulses to the ventricles and are responsible for the early afterdepolarizations that not only are associated with long QT syndrome, but that also occur upon exposure to drugs that prolong cardiac repolarization [10–13]. In particular, canine Purkinje fibers have become an accepted model for investigating the effects of pharmaceutical compounds on action potential duration (APD) because of their sensitivity to agents that demonstrate prolongation of APD [14–16]. Studies have suggested that drug-induced prolongation of the AP in canine Purkinje fibers correlates with a drug's ability to induce a clinically relevant increase in human QT interval that may be predictive of Torsades de Pointes [14,15].

4-Aminopyridine (fampridine) is a potassium channel blocker that significantly improves walking speed relative to placebo in a proportion of patients with multiple sclerosis [17–20]. The proposed mechanism of action is a dose-dependent blockade of voltage-gated potassium channels that occurs at clinically relevant concentrations (0.2 to 2 µM; 18 to 180 ng/mL) [21,22]. As suggested *in vitro* using demyelinated axons, the observed clinical effects with 4-aminopyridine occur as a result of an increased ratio between the action current generated by the impulse and the minimum amount of action current needed to maintain axonal conduction across demyelinated internodes [23]. Additionally, 4-aminopyridine enhances neurotransmission in intact neurons, possibly by increasing calcium influx at presynaptic sites [24]; however, the role of this effect as a contributory factor to the clinical efficacy of the agent is unclear.

Since 4-aminopyridine affects potassium channels, it is important to evaluate the cardiac safety of the agent with respect to changes in AP that could result in arrhythmias and Torsades de Pointes. As part of the drug evaluation process for 4-aminopyridine and in accordance with recent guidelines, preclinical testing of 4-aminopyridine was undertaken to determine the potential of the agent for inducing clinically significant QT prolongation. A previous preclinical study that evaluated the *in vitro* effects of 4-aminopyridine on the hERG channel current expressed in a human cell line reported a low potential of the drug for QT interval prolongation [25]. In the current study, we evaluated the ability of 4-aminopyridine to alter the AP parameters of isolated canine Purkinje fibers, which can also provide an indication of its ability to induce QT prolongation.

## Methods

The study protocol was reviewed and approved by the Institutional Animal Care and Use Committee. This study was performed in accordance with the current guidelines for animal welfare (Animal Welfare Act, the Guide for the Care and Use of Laboratory Animals, and the Office of Laboratory Animal Welfare). The study center is registered with the US Department of Agriculture (USDA) and is fully accredited by the Association for Assessment and Accreditation of Laboratory Animal Care International; all animals were individually housed in compliance with USDA guidelines.

Adult male beagle dogs (N = 5) ranging in weight from 7.0 to 10.0 kg, obtained from Marshall Farms (North Rose, NY, USA), were housed individually and provided food up to 400 g/day and water *ad libitum* until use. At the time of experimentation, animals were anesthetized with sodium pentobarbital and euthanized by cardiac excision. After removal of the heart, Purkinje fiber bundles were dissected from the left or right ventricle. Individual Purkinje fibers, each from a different experimental animal, were then isolated and mounted in a recording chamber with continuous perfusion of oxygenated (95% O_2_/5% CO_2_) Tyrode's solution (NaCl 125 mM, KCl 4 mM, NaHCO_3_ 25 mM, MgCl_2_ 1 mM, NaH_2_PO_4_ 1.2 mM, CaCl_2_ 1.8 mM, glucose 5.5 mM) at a rate of approximately 5 mL/minute. The bath temperature was maintained at 35°C to 38°C and the pH at 7.3 to 7.5.

The fibers were electrically stimulated at 1.0 Hz using bipolar silver electrodes, and each Purkinje fiber was impaled with a KCl (3M) filled glass microelectrode (resistance range, 16 to 26 MΩ) to monitor transmembrane potential. Purkinje fibers were allowed to equilibrate for 60 minutes to achieve stable AP prior to experimentation. The AP was continuously monitored and recorded using an Axoclamp-2B amplifier (Axon Instruments; Union City, CA, USA) with a Notocord-hem 3.4 data capture system (Notocord Systems; Croissy sur Seine, France). The recorded AP parameters included resting membrane potential (RMP); overshoot (OS); amplitude of AP (APA); maximal rate of depolarization of the AP upstroke (V_max_); and APD at 30%, 50%, and 90% repolarization (APD_30_, APD_50_, and APD_90_, respectively).

Following the establishment of stable AP, pretreatment control values at 1.0 Hz were defined by insertion of an event marker, pacing frequency was reduced to 0.5 Hz, and another event marker was inserted to define the pretreatment control values after stabilization at 0.5 Hz. Pacing frequency was then returned to 1.0 Hz, and the fibers were equilibrated with 4-aminopyridine 0.5 µM for at least 25 minutes, with measurement of electrophysiologic responses subsequently recorded at 1.0 and 0.5 Hz (5 to 10 minutes). The process was repeated for 4-aminopyridine 5 µM, 50 µM, and 500 µM, with final flushing and washout to evaluate recovery. Each fiber was evaluated separately, as indicated above.

Individual parameter data are presented as the mean (± standard error [SE]) of AP values recorded from all fibers during a 60-second period following the corresponding event marker. Since each fiber served as its own control, changes in APD_30_, APD_50_, and APD_90_ can be expressed as percent change from baseline. Analysis of variance, followed by Tukey's HSD (honestly significant difference) Multiple Comparison Test, was used to compare treatment effects with control values (Systat version 9.01; Systal Software, Inc., Chicago, IL); *P* values ≤ 0.05 were considered statistically significant.

## Results

**Figure 1:**
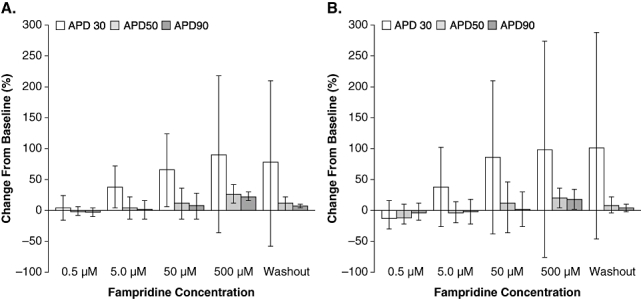
Effect of 4-aminopyridine on action potential duration (APD) at 30% (APD_30_), 50% (APD_50_), and 90% (APD_90_) repolarization. Data expressed as mean percent of control values (± 95% confidence interval). (a) 1.0-Hz stimulation frequency; (b) 0.5-Hz stimulation frequency.

**Figure 2:**
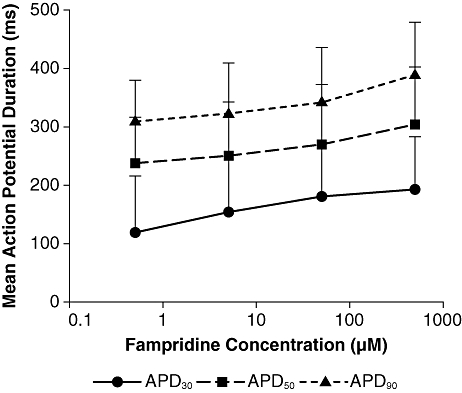
The dose-response effect of 4-aminopyridine on action potential duration (APD) at 30% (APD_30_), 50% (APD_50_), and 90% (APD_90_) repolarization. Data expressed as mean action potential duration with upper bound of 95% confidence interval.

Four Purkinje fibers were used to evaluate the effect of 4-aminopyridine on AP; a fifth fiber was not used because of unstable AP recordings associated with microelectrode impalement problems. Table 1 and Figure 1 present the mean values for APA, OS, RMP, and V_max_ for all fampridine concentrations at 0.5 and 1 Hz, and Table 2 shows the corresponding mean values for APD_30_, APD_50_, and APD_90_. The concentration effect, or dose response, curves are presented in Figure 2.

**Table 2:** Mean (±standard error) values of action potential duration (APD) at 30% (APD_30_), 50% (APD_50_), and 90% (APD_90_) repolarization in isolated dog Purkinje fibers at 1.0 and 0.5 Hz

	APD_30_ (ms)		APD_50_ (ms)		APD_90_ (ms)	
Treatment (n)	1 Hz	0.5 Hz	1 Hz	0.5 Hz	1 Hz	0.5 Hz
Control (4)	118.5 ± 32.0	124.2 ± 40.2	242.1 ± 26.2	276.5 ± 33.8	319.6 ± 22.6	367.6 ± 28.8
Fampridine 0.5 µM (4)	118.6 ± 29.9	114.1 ± 32.4	237.6 ± 24.9	259.3 ± 30.7	310.6 ± 21.4	349.2 ± 23.4
Fampridine 5 µM (4)	153.0 ± 31.0	148.6 ± 33.1	250.8 ± 28.6	267.2 ± 32.1	323.9 ± 26.5	361.5 ± 32.5
Fampridine 50 µM (4)	180.2 ± 34.2	193.5 ± 42.1	269.3 ± 31.7	293.2 ± 41.7	342.2 ± 29.7	373.6 ± 38.9
Fampridine 500 µM (4)	192.1 ± 27.7	186.7 ± 29.1	303.5 ± 31.4	331.5 ± 38.5	390.0 ± 28.2	434.5 ± 39.0
Washout	176.0 ± 26.4	191.1 ± 32.0	266.8 ± 22.8	296.3 ± 26.4	340.6 ± 22.8	381.8 ± 27.9

**Table 1:** Mean (±standard error) values of action potential parameters in isolated dog Purkinje fibers at 1.0 and 0.5 Hz

	RMP (mV)		OS (mV)		APA (mV)		V_max_ (mV/ms)	
Treatment (N)	1 Hz	0.5 Hz	1 Hz	0.5 Hz	1 Hz	0.5 Hz	1 Hz	0.5 Hz
Control (4)	−97.4 ± 1.5	−94.4 ± 1.7	31.5 ± 0.6	31.5 ± 0.6	129.0 ± 1.5	125.9 ± 2.1	658.4 ± 70.0	657.5 ± 69.5
Fampridine 0.5 µM (4)	−96.7 ± 1.5	−90.7 ± 1.2	31.5 ± 1.0	30.8 ± 1.4	128.3 ± 2.1	121.6 ± 1.9	700.0 ± 92.6	616.6 ± 51.9
Fampridine 5 µM (4)	−96.0 ± 1.5	−90.5 ± 2.6	28.4 ± 0.5	27.5 ± 1.3	124.3 ± 1.8	118.0 ± 3.7	612.8 ± 70.8	584.1 ± 83.5
Fampridine 50 µM (4)	−97.1 ± 1.3	−92.7 ± 0.4	27.0 ± 2.0	26.6 ± 3.1	124.2 ± 2.7	119.3 ± 2.9	630.7 ± 76.5	611.9 ± 70.3
Fampridine 500 µM (4)	−97.1 ± 1.7	−91.3 ± 1.5	26.5 ± 3.9	27.9 ± 3.9	123.6 ± 5.4	119.2 ± 5.2	566.3 ± 87.1	570.3 ± 81.5
Washout	−98.1 ± 1.0	−95.1 ± 1.0	28.1 ± 2.4	28.6 ± 2.5	126.2 ± 3.2	123.7 ± 3.4	588.1 ± 55.2	600.3 ± 55.4

APA = amplitude of action potential; OS = overshoot; RMP = resting membrane potential; V_max_ = maximal rate of depolarization of the action potential upstroke.

At the 1.0-Hz stimulation frequency, there appeared to be a dose-dependent increase in APD at 5, 50 and 500 µM at all three repolarization thresholds (30%, 50%, and 90%) (Figure 1a). However, only the 4-aminopyridine 500-µM concentration resulted in increased AP durations that were statistically significant (*P* < 0.05). As shown in Figure 1b, the proportional increases from baseline (95% confidence intervals) for the 500 µM concentration were 90.8% (-36.4%, 218.0%), 25.8% (11.9%, 39.7%), and 22.0% (14.9%, 29.1%) for APD_30_, APD_50_, and APD_90_, respectively. Although similar increases compared with baseline were observed at the 0.5-Hz stimulation frequency (Figure 2), 98.8% (−75.9%, 273.5%), 20.5% (4.9%, 36.1%), and 18.1% (2.5%, 33.7%) higher than baseline values for APD_30_, APD_50_, and APD_90_, respectively. Some dose-dependency was observed with respect to the effect of 4-aminopyridine on APD at the 0.5-Hz frequency, especially at the lower 4-aminopyridine concentrations, but this effect was not as marked as at 1.0 Hz.

**Figure 3:**
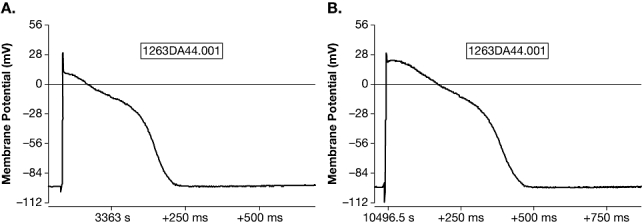
Representative tracings of action potential in isolated canine Purkinje cells in the absence (a) and presence (b) of 4-aminopyridine 500 µM at a stimulation frequency of 1.0 Hz. Horizontal line denotes 0 mV.

Representative tracings of the AP triggered by field stimulation at 1.0 Hz in an isolated control Purkinje fiber under control conditions (Figure 3a) and after the addition of 4-aminopyridine 500 µM (Figure 3b) demonstrate slowing of repolarization, with an increase in the height of the plateau and a significantly prolonged AP relative to the control.

## Discussion

This study reports the effects of 4-aminopyridine on the AP in a small sample size of isolated dog Purkinje fibers. A significant effect was observed at 500 µM 4-aminopyridine (*P* < 0.05), and an apparent dose-dependent increase was observed in APD_30_ at 0.5, 5.0, and 50 µM. An increase could potentially be attributed to the effects of 4-aminopyridine on the transient outward potassium current (I_to_). In Kenyon and Gibbons' [26] early work on action potential and membrane currents in sheep Purkinje cells, they reported that 4-aminopyridine at concentrations between 0.01 and 0.5 mM slowed the rate of phase 1 repolarization and shifted the plateau of the action potential to less negative potentials. These effects were attributed to a 4-aminopyridine-sensitive component of the early outward current. It was subsequently shown that the I_to_ of canine cardiac Purkinje cells is inhibited by 4-aminopyridine within the range of concentrations used in the current study, with a reported IC_50_ (half maximal [50%] inhibitory concentration) of 50 µM [27]. A study of 4-aminopyridine in a canine model reported that even at the maximum plasma concentration that was achieved *in vivo* by drug infusion (250 uM), ventricular and atrial I_to_ were not significantly affected [28]. Studies characterizing repolarizing currents in cardiac Purkinje cells from other species (sheep and rabbit) have also reported an effect of 4-aminopyridine on the I_to_, albeit at higher 4-aminopyridine concentrations (0.5 to 2.0 mM) [29,30], possibly due to interspecies differences in the ion channels. However, I_to_ is primarily associated with early phase 1 repolarization [31] and, although it is a key regulator of this phase of ventricular repolarization, it does not affect the total APD of canine ventricular myocytes, and thus does not contribute to the critical end point of T wave or ventricular APD dispersion [32].

A number of other studies have investigated the role of 4-aminopyridine and APD in Purkinje fibers and other cardiac tissue. Sridhar et al [33] demonstrated that 4-aminopyridine at 50 and 100 µM significantly prolonged APD in canine ventricular myocytes at APD50 and 90 (1 Hz), and voltage clamp experiments suggested that a 4-aminopyridine-sensitive rapidly activating plateau outward current is present in left ventricular canine myocytes. However, regional distinctions at the molecular level in canine repolarizing currents may explain differences in cellular responsiveness; for example, under voltage clamp conditions, phenylephrine had no effect on I_to_ in canine epicardial myocytes, whereas Purkinje cell I_to_ was inhibited by the same treatment [34].

4-aminopyridine 500 µM significantly increased the APD at all three polarization levels, but only at the 1.0-Hz stimulation frequency although numerically the changes with 0.5-Hz were of similar magnitude. The rapidly activating component of the I_Kr_ is not the only the primary outward current responsible for initiation of final AP repolarization in canine ventricular muscle and Purkinje fibers [35], but its inhibition is a common cause of AP prolongation leading to the development of Torsades de Pointes [7,36,37].

The major channel protein of I_Kr_ is encoded by hERG, which is primarily responsible for the final repolarization phases of the ventricular AP [2,8,9]. When this gene is stably transfected into an expression system, such as human embryonic kidney cells, expression of hERG produces a current with the same characteristics of I_Kr_[38]. Since many of the drugs associated with induction of Torsades de Pointes have also been reported to inhibit hERG [6,7], it is important to note that our results are consistent with an *in vitro* study showing that inhibition of hERG channel current in stably transfected human embryonic kidney cells was concentration-dependent between 4-aminopyridine concentrations of 0.3 and 30 mM [25]. However, two factors need to be considered in determining the clinical relevance of the *in vitro* effects of 4-aminopyridine. The first factor is that reverse rate dependence—that is, loss of effect at greater frequency, which is characteristic of most drugs that predominantly block the I_Kr_ repolarizing current—was not observed in the currentstudy. The lack of reverse rate dependence suggests that inhibition of ion channels other than hERG may contribute to the 4-aminopyridine-associated AP prolongation. The second factor is that drugs associated with Torsades de Pointes generally inhibit hERG at concentrations approximating therapeutic plasma concentrations [7]. Average plasma concentrations range from 0.243 ± 0.113 µM during steady state at the therapeutic dose of 10 mg twice daily to 0.974 ± 0.451 µM at four times the therapeutic dose [18].

A limitation of the study is that the analysis was based solely on the measurement of the AP parameters without measurement of current. The small sample size is a further limitation of this study that should be considered when interpreting the results. No *a priori* power calculations were performed; however, post hoc it would appear that with a sample size of 4, the power to detect a significant difference in APD change of approximately 65% was 70%. The lack of statistical significance for some of the analyses may reflect inter-preparation variability that may have been accentuated by the small sample size. Nevertheless, this *in vitro* electrophysiologic study suggests that 4-aminopyridine resulted in AP prolongation in a small sample of canine Purkinje fibers. A larger sample size would better define the concentration effect relationship.

## References

[1] Fenichel RR, Malik M, Antzelevitch C, Sanguinetti M, Roden DM, Priori SG (2004). Drug-induced torsades de pointes and implications for drug development. J Cardiovasc Electrophysiol.

[2] Sanguinetti MC, Keating MT (1997). Role of delayed rectifier potassium channels in cardiac repolarisation and arrhythmias. News Physiol Sci.

[3] US Food and Drug Administration (2005). Guidance for industry: E14 clinical evaluation of QT/qtc interval prolongation and proarrhythmic potential for non-antiarrhythmic drugs.

[4] International Conference on Harmonisation of Technical Requirements for Registration of Pharmaceuticals (ICH) (2005). ICH harmonised tripartite guideline: The clinical evaluation of QT/qtc interval prolongation and proarrhythmic potential for non-antiarrhythmic drugs [Internet].

[5] US Food and Drug Administration [Internet] (2004). ICH S7B Guideline. http://www.fda.gov/cder/guidance/5533dft.htm.

[6] Katchman AN, Koerner J, Tosaka T, Woosley RL, Ebert SN (2006). Comparative evaluation of HERG currents and QT intervals following challenge with suspected torsadogenic and nontorsadogenic drugs. J Pharmacol Exp Ther.

[7] Redfern WS, Carlsson L, Davis AS, Lynch WG, MacKenzie I, Palethorpe S (2003). Relationships between preclinical cardiac electrophysiology, clinical QT interval prolongation and torsade de pointes for a broad range of drugs: Evidence for a provisional safety margin in drug development. Cardiovasc Res.

[8] Sanguinetti MC, Jiang C, Curran ME, Keating MT (1995). A mechanistic link between an inherited and an acquired cardiac arrhythmia: HERG encodes the IKr potassium channel. Cell.

[9] Trudeau MC, Warmke JW, Ganetzky B, Robertson GA HERG, a human inward rectifier in the voltage-gated potassium channel family. Science.

[10] el-Sherif N, Caref EB, Yin H, Restivo M (1996). The electrophysiological mechanism of ventricular arrhythmias in the long QT syndrome. Tridimensional mapping of activation and recovery patterns. Circ Res.

[11] el-Sherif N, Zeiler RH, Craelius W, Gough WB, Henkin R (1988). QTU prolongation and polymorphic ventricular tachyarrhythmias due to bradycardia-dependent early afterdepolarizations. Afterdepolarizations and ventricular arrhythmias. Circ Res.

[12] Nattel S, Quantz MA (1988). Pharmacological response of quinidine induced early after depolarisations in canine cardiac Purkinje fibres: Insights into underlying ionic mechanisms. Cardiovasc Res.

[13] Roden DM (1993). Early after-depolarizations and torsade de pointes: Implications for the control of cardiac arrhythmias by prolonging repolarization. Eur Heart J.

[14] Champeroux P, Viaud K, El Amrani AI, Fowler JS, Martel E, Le Guennec JY (2005). Prediction of the risk of Torsade de Pointes using the model of isolated canine Purkinje fibres. Br J Pharmacol.

[15] Gintant GA, Limberis JT, McDermott JS, Wegner CD, Cox BF (2001). The canine Purkinje fiber: An in vitro model system for acquired long QT syndrome and drug-induced arrhythmogenesis. J Cardiovasc Pharmacol.

[16] Gralinski MR (2003). The dog's role in the preclinical assessment of QT interval prolongation. Toxicol Pathol.

[17] Goodman AD, Brown TR, Cohen JA, Krupp LB, Schapiro R, Schwid SR (2008). Dose comparison trial of sustained-release fampridine in multiple sclerosis. Neurology.

[18] Goodman AD, Cohen JA, Cross A, Vollmer T, Rizzo M, Cohen R (2007). Fampridine-SR in multiple sclerosis: A randomized, double-blind, placebo-controlled, dose-ranging study. Mult Scler.

[19] Polman CH, Bertelsmann FW, van Loenen AC, Koetsier JC (1994). 4-aminopyridine in the treatment of patients with multiple sclerosis. Long-term efficacy and safety. Arch Neurol.

[20] Schwid SR, Petrie MD, McDermott MP, Tierney DS, Mason DH, Goodman AD (1997). Quantitative assessment of sustained-release 4-aminopyridine for symptomatic treatment of multiple sclerosis. Neurology.

[21] Judge SI, Bever CT (2006). Potassium channel blockers in multiple sclerosis: Neuronal Kv channels and effects of symptomatic treatment. Pharmacol Ther.

[22] Stuhmer W, Ruppersberg JP, Schroter KH, Sakmann B, Stocker M, Giese KP (1989). Molecular basis of functional diversity of voltage-gated potassium channels in mammalian brain. EMBO J.

[23] Targ EF, Kocsis JD (1985). 4-Aminopyridine leads to restoration of conduction in demyelinated rat sciatic nerve. Brain Res.

[24] Lundh H (1978). Effects of 4-aminopyridine on neuromuscular transmission. Brain Res.

[25] Renganathan M, Sidach S, Blight AR (2009). Effects of 4-aminopyridine on cloned hERG channels expressed in mammalian cells. Arch Drug Inf.

[26] Kenyon JL, Gibbons WR (1979). 4-Aminopyridine and the early outward current of sheep cardiac Purkinje fibers. J Gen Physiol.

[27] Han W, Wang Z, Nattel S (2000). A comparison of transient outward currents in canine cardiac Purkinje cells and ventricular myocytes. Am J Physiol Heart Circ Physiol.

[28] Nattel S, Matthews C, De Blasio E, Han W, Li D, Yue L (2000). Dose-dependence of 4-aminopyridine plasma concentrations and electrophysiological effects in dogs. Potential relevance to ionic mechanisms in vivo. Circulation.

[29] Boyett MR (1981). Effect of rate-dependent changes in the transient outward current on the action potential in sheep Purkinje fibres. J Physiol.

[30] Cordeiro JM, Spitzer KW, Giles WR (1998). Repolarizing K+ currents in rabbit heart Purkinje cells. J Physiol.

[31] Patel SP, Campbell DL (2005). Transient outward potassium current, “Ito”, phenotypes in the mammalian left ventricle: Underlying molecular, cellular and biophysical mechanisms. J Physiol.

[32] Sun X, Wang HS (2005). Role of the transient outward current (Ito) in shaping canine ventricular action potential: A dynamic clamp study. J Physiol.

[33] Sridhar A, da Cunha DN, Lacombe VA, Zhou Q, Hamlin RL, Carnes CA (2007). The plateau outward current in canine ventricle, sensitive to 4-aminopyridine, is a constitutive contributor to ventricular repolarization. Br J Pharmacol.

[34] Robinson RB, Liu QY, Rosen MR (2000). Ionic basis for action potential prolongation by phenylephrine in canine epicardial myocytes. J Cardiovasc Electrophysiol.

[35] Varro A, Balati B, Iost N, Takacs J, Virag L, Lathrop DA (2000). The role of the delayed rectifier component IKs in dog ventricular muscle and Purkinje fibre repolarization. J Physiol.

[36] Nattel S (1999). The molecular and ionic specificity of antiarrhythmic drug actions. J Cardiovasc Electrophysiol.

[37] Witchel HJ (2007). The hERG potassium channel as a therapeutic target. Expert Opin Ther Targets.

[38] Zhou Z, Gong Q, Ye B, Fan Z, Makielski JC, Robertson GA (1998). Properties of HERG channels stably expressed in HEK 293 cells studied at physiological temperature. Biophys J.

